# Explaining variation in Down’s syndrome screening uptake: comparing the Netherlands with England and Denmark using documentary analysis and expert stakeholder interviews

**DOI:** 10.1186/1472-6963-14-437

**Published:** 2014-09-25

**Authors:** Neeltje MTH Crombag, Ynke E Vellinga, Sandra A Kluijfhout, Louise D Bryant, Pat A Ward, Rita Iedema-Kuiper, Peter CJI Schielen, Jozien M Bensing, Gerard HA Visser, Ann Tabor, Janet Hirst

**Affiliations:** Department of Obstetrics, University Medical Centre Utrecht, Huispost KE 04.123.1, Postbus 85090, 3508 AB Utrecht, The Netherlands; Erasmus University Rotterdam, Rotterdam, The Netherlands; Leeds Institute of Health sciences, Faculty of Medicine and Health, University of Leeds, Leeds, UK; NHS Fetal Anomaly Screening Programme, Innovation Centre, University of Exeter, Exeter, UK; National Institute for Public Health and the Environment, Diagnostic Laboratory for Infectious Diseases and Perinatal Screening Bilthoven, Bilthoven, The Netherlands; The Netherlands Institute for Health Services Research Utrecht, Utrecht, The Netherlands; Center for Fetal Medicine, Departmentof Obstetrics and Gynecology, Copenhagen University Hospital, Rigshospitalet, Kragujevac, Denmark

**Keywords:** Down’s syndrome, Antenatal screening, Utilization of healthcare, Uptake, International comparison

## Abstract

**Background:**

The offer of prenatal Down’s syndrome screening is part of routine antenatal care in most of Europe; however screening uptake varies significantly across countries. Although a decision to accept or reject screening is a personal choice, it is unlikely that the widely differing uptake rates across countries can be explained by variation in individual values alone.

The aim of this study was to compare Down’s syndrome screening policies and programmes in the Netherlands, where uptake is relatively low (<30%) with England and Denmark where uptake is higher (74 and > 90% respectively), in an attempt to explain the observed variation in national uptake rates.

**Methods:**

We used a mixed methods approach with an embedded design: a) documentary analysis and b) expert stakeholder analysis. National central statistical offices and legal documents were studied first to gain insight in demographic characteristics, cultural background, organization and structure of healthcare followed by documentary analysis of primary and secondary sources on relevant documents on DSS policies and programme. To enhance interpretation of these findings we performed in-depth interviews with relevant expert stakeholders.

**Results:**

There were many similarities in the demographics, healthcare systems, government abortion legislation and Down’s syndrome screening policy across the studied countries. However, the additional cost for Down’s syndrome screening over and above standard antenatal care in the Netherlands and an emphasis on the ‘right not to know’ about screening in this country were identified as potential explanations for the ‘low’ uptake rates of Down’s syndrome screening in the Netherlands. The social context and positive framing of the offer at the service delivery level may play a role in the relatively high uptake rates in Denmark.

**Conclusions:**

This paper makes an important contribution to understanding how macro-level demographic, social and healthcare delivery factors may have an impact on national uptake rates for Down’s syndrome screening. It has suggested a number of policy level and system characteristics that may go some way to explaining the relatively low uptake rates of Down’s syndrome screening in the Netherlands when compared to England and Denmark.

**Electronic supplementary material:**

The online version of this article (doi:10.1186/1472-6963-14-437) contains supplementary material, which is available to authorized users.

## Background

The offer of prenatal Down’s syndrome screening (DSS) is part of routine antenatal care in most of the European Union. First trimester combined Down’s syndrome screening (DSS) is now the most used approach and combines biochemical maternal serum screening and an ultrasound fetal nuchal translucency measurement to calculate a pregnancy specific risk factor. Following a ‘high risk’ result, for example higher than 1:200, diagnostic testing is offered as an option. Following a positive diagnosis of Down’s syndrome termination of pregnancy is offered as an option in those countries were abortion is legal for fetal abnormalities. Prenatal testing can therefore present social, emotional and social dilemmas and it is generally accepted that parent’s decision to accept or decline the offer of DSS should be informed by parental values and made autonomously, that is without direction from health professionals.

**Figure 1:**
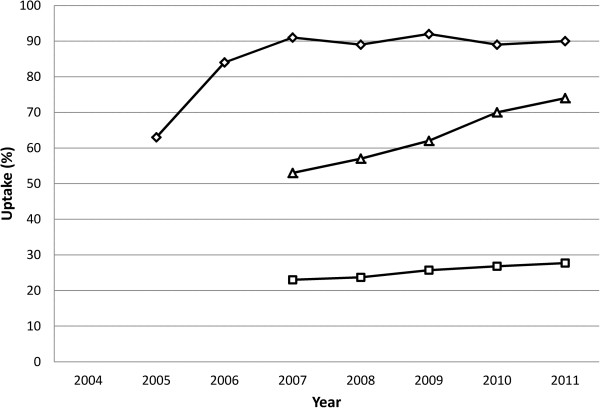
**Introduction and trends of uptake of first trimester combined screening in Denmark (diamonds), England (triangles) and the Netherlands (squares).**

A review of early ‘demonstration’ projects in the 1990s, conducted to assess the practicality and acceptability of DSS suggested that women in different countries tended to make similar decisions about accepting DSS [1]. Across different types of tests in 17 studies in the USA and Europe, average DSS uptake rates of around 80% were reported. This also included findings from one study in the Netherlands [2]. Once DSS programmes were implemented within national health care systems, however, uptake rates have shown to vary significantly across countries [3, 4]. In the Netherlands, for example, DSS uptake has been found to be low (<30%) when compared to other northern European countries (74% in England and ≥ 90% in Denmark) (Figure 1) [5–9]. These differences may be considered surprising, given the findings from the early demonstration projects and the apparently close resemblance in cultural, social and healthcare factors across these countries and, hence, they require explanation.

Personal decisions to accept or decline DSS are known to be influenced by a range of psychosocial factors including health beliefs and attitudes towards disability and termination of pregnancy [10]. Certain social cultural characteristics mediate these factors, for example, uptake rates of DSS are lower in some minority ethnic groups [11–13] and religious observance is an important factor for some women choosing not to use DSS [14, 15]. Hence variation in uptake rates may reflect differences in individual choice, which then manifest in the regional population in which the screening service is located. However, highly significant variation in screening uptake between close geographical locations in the UK, for example, suggests that service delivery factors and the healthcare context in which individual decisions are made also play an important part in these differences [16]. Service delivery and social structural differences may also play a part in explaining the wide variation in uptake across European countries; differences which were not anticipated by the early demonstration projects. The macro level influence that screening policies might have on uptake at a national level has also not yet been investigated at all.

The aim of this study was to consider whether structural and policy differences between the Netherlands, England and Denmark could help explain the observed variation in national uptake rates. Our research questions were to identify similarities and differences in these three countries across a) the socio-demographic population profiles, b) cultural factors, organization and structure of health and social care systems and c) DSS policies and programme characteristics.

## Methods

This study adopted a pragmatic qualitative approach to help describe and explain relationships. To answer the research questions we used a mixed methods approach with an embedded design: a) analysis of relevant documents to describe differences and similarities within established policies and programmes and b) to further explore this information we performed in depth interviews with relevant experts [17]. First, we studied central statistical offices and legal documents to gain insight in demographic characteristics, cultural background, organization and structure of healthcare in each of the three countries. Second, we performed documentary analysis of primary and secondary sources on relevant documents on DSS policies and programme, and third, to enhance interpretation of these findings we performed in-depth interviews with relevant expert stakeholders in the participating countries to provide insight in initial intentions and formal rules of the national DSS policy and the impact on performance [18, 19].

### National statistics and legal documents

Demographic characteristics, cultural background, organization and structure of healthcare in each of the three countries were obtained from central statistical offices and legal documents. Legal documents were direct products of national Government and Ministries. Secondary sources were used to supplement these findings (Additional file 1).

### Documentary analysis

Documentary analysis can be described as the analysis of documents that contain information about the phenomenon to be studied [20]. In order to identify differences and similarities regarding DSS policies and programme characteristics we performed documentary analysis on both primary and secondary sources regarding DSS policies and characteristics (Additional file 2).

#### Sample and procedure

First, individual prenatal DSS pathways were described in each of three countries. Secondly, documents which underpinned the different steps in these pathways were purposively sampled. Documents were assessed for criteria on relevance, reliability and origin, that is published or approved by national screening organisations, national government or national health boards. For example, in the Netherlands, within the individual pathway DSS, there is a ‘step’ in which women have a right to refuse information. Documents underlying this specific ‘step’ were searched in National screening programme databases, websites, legal websites, governmental websites and assessed for criteria of reliability and origin. Documents underpinning this ‘step’ are based on an interpretation of the principle of autonomy and is incorporated in the screening programme [21–23].

The sample of documents was obtained from National screening databases (both websites and upon request), governmental websites, national Health Council, national Health board, ministries, legal websites, professional websites and national health services websites. Secondary sources were obtained from peer-reviewed literature to supplement findings [24, 18]. The quality control criteria of authenticity, credibility, representatives, and meaning were applied on selected documents and subsequently selected for content analysis [25]. With the sample of documents underlying the individual pathways we intended to design an overview of the ‘screening environment’ in which individuals participate in DSS in the countries studied.

We used content analysis to elucidate categories regarding availability, provision and access to DSS in the three countries. These categories were used to compare national healthcare policies.

### Stakeholder interviews

#### Sample

Expert stakeholders were defined as persons having a specific authority on the subject of DSS; holding a position of leadership or influence in the development, organisation or delivery of DSS screening organization [19]. Expert stakeholders were anticipated to include those with a scientific background or professional specialism in prenatal screening, or those involved in DSS related policy-making. Purposive sampling of expert stakeholders using snowballing techniques identified 15 people for interview; four were based in the Netherlands, five in England and six in Denmark. Snowball sampling is commonly used where expert knowledge is the prerequisite for inclusion in a sample. The research starts with a small number of known stakeholders who then identify other important stakeholders who meet the study inclusion criteria [26]. In this study we started with three initial informants; persons holding a leading position in the national DSS programme (the Netherlands and England) or leading position of influence in the development in DSS (Denmark). The initial informant than nominated the participants in their country who met the eligibility criteria for the study. In the Netherlands two professional specialists were identified, and an expert involved in policy making and development. In Denmark, three professional specialists were identified, of which two were also involved in research. Besides an expert in policy making and an expert on development and research were identified. In England two professional specialists were identified, both also involved in development. Furthermore an expert in research was identified and one involved in development of the programme.

#### Development of the interview schedule

Prior to the interviews the researchers explicitly discussed their personal views about DSS to reflect on how subjective and intersubjective elements could influence data collection, analysis and interpretation [19, 27].

The interview schedule was informed by data from the documentary analysis, and part of the interviews were concerned with validating data found in the documentary analysis, checking on apparent inconsistencies or ‘filling in’ missing data identified [19]. After performing a pilot interview, the researchers discussed the interview schedule, disagreements were discussed until consensus was reached.

#### Ethical approval

Ethical approval for this study was obtained for participants employed by the National Health Service via the National Research Ethics Service in England (reference number 11/NE/0166). In Denmark (http://www.cvk.sum.dk/English/actonabiomedicalresearch.aspx) and the Netherlands (http://www.ccmo.nl/en/non-wmo-research) ethical approval was not required for research involving staff only.

#### Interview procedure

The interviews were performed at the interviewee’s workplace. Two researchers were present at each interview (SK, YV) [19]. The interviews were undertaken in English in England and Denmark, and in Dutch in the Netherlands. With the permission of participants, the interviews were digitally recorded.

#### Analysis

All interviews were transcribed verbatim by the researchers. After transcription, the interviewees were given the chance to respond to the interview transcription on accuracy only. The researchers read the transcripts and discussed first impressions, thoughts and initial analysis. The researchers identified themes in the transcripts and emphasized on value of informed choice, influence on screening choices and the impact of financial considerations [28]. The Dutch data were analysed first and subsequently translated into English.

The transcriptions were coded and stored on a university firewall protected secure server, accessible via password for security and safety, prior to electronic transfer to Utrecht Medical Centre for access and storage in the Netherlands. Data were removed from audio recorder and computer servers in Denmark and England once safe transfer was completed and secured.

## Results

### Documentary analysis

#### Population characteristics, health and social care

Table 1 summarises the known basic demographic data of the three countries. Birth rates, maternal age, percentage of the population born outside each country, and rates of tertiary education were similar across countries. Reported religious affiliation was substantially lower in the Netherlands, although measures of religious observance were not available. Gross Domestic Product (GDP) was lowest in the UK but all three countries were highly economically developed and in the top quartile of the United Nation’s Human Development Index.

**Table 1:** **Population characteristics**

	The Netherlands	United Kingdom*	Denmark
**Birth rate per 1000, 2010**	11.1	13.0	11.4
**Maternal age, 1st child, 2010**	29.4	29.5	29.1
**Reported religious affiliation (2009)**	56%	85%	80%
**Foreign-born population (2008)**	10,9%	11%	8.8%
	2.5% EU	3.5% EU	2.6% EU
	8.4% Non EU	7.5% Non EU	6.2% Non EU
**% of population aged 30–34 yrs with tertiary education qualifications (2009)**	35.8	37.9	41.2
GDP per capita in PPS**	133	112	127

*Data for England only available at United Kingdom level i.e. the four countries including England.

**Gross Domestic Product in Purchasing Power Standards.

Table 2 summarises some key information about health and social care systems including legislation available for disabled people and their families.

**Table 2:** **Health and social care legislation and funding**

	The Netherlands	United Kingdom*	Denmark
**Healthcare system**	Social Security Healthcare system	National Health Service	National Healthcare system
**Funding of healthcare system**	Earmarked premiums	General taxation	General taxation
**Organisation healthcare system**	Health care provided by non-profit hospitals and individual practitioners	Stated owned hospitals and general practitioners have contracts with NHS	State owned hospitals and general practitioners have contracts with NHS
	Strong influence of healthcare providers and (social) insurers	Strong influence of state: SSSMinister of Health responsible for budget	Strong influence of state: Minister of Health responsible for budget
	Government responsible for accessibility, affordability and quality of healthcare		
**Legislation regarding equality for disabled individuals**	Act on Equal Treatment of Disabled and Chronically Ill People (2003) (WGBH/CZ)	Equality Act (2010)	Parliamentary Resolution on equal opportunities for and equal treatment of people with disabilities (1993) (BSF 43)
	*Wet gelijke behandeling op grond van handicap of chronische ziekte*		*Folketingsbeslutning om ligestilling og ligebehandling af handicappede med andre borgere*

*Data for England only available at United Kingdom level i.e. the four countries including England.

Equal access to basic healthcare services was guaranteed by all systems and monitored by the national governments. Although healthcare systems could be categorised differently, National Healthcare Services (Denmark and England) and Social Security Healthcare system (the Netherlands), all three countries provided a relatively high level of social and health care for disabled individuals when compared to lower income countries, although service coverage was not assessed. Across all countries financial provision and services for disabled people aimed to support the principle of equal rights and equality of opportunities in concordance with the United Nations Standard Rules on the Equalization of Opportunities for Persons with Disabilities [29–32].

Table 3 summarises legislation relevant to termination of pregnancy in the three countries. Termination of pregnancy (TOP) for social reasons was legal in all the three countries studied and at no cost at point of delivery. The gestational age at which a TOP can legally be performed differed between countries. Denmark was the most restrictive in terms of gestational limits. The UK was the most liberal in terms of termination for fetal anomaly with no upper gestation limit where “there is a substantial risk that if the child were born it would suffer from such physical or mental abnormalities as to be seriously handicapped”[33].

**Table 3:** **Abortion legislation and funding**

	The Netherlands	United Kingdom*	Denmark
**Termination of pregnancy (TOP) for social reasons**	Legal until 24 weeks gestation	Legal until 24 weeks gestation	Legal until 12 weeks gestation
**TOP for fetal anomalies**	After 24 weeks only in very limited cases	No gestational limit if there is a substantial risk the child would suffer from such physical or mental abnormality as to be seriously handicapped	Permitted up to 22 weeks if there is a substantial risk that the child would suffer from severe mental or physical abnormality
**Payment for TOP**	Free at the point of delivery	Free at the point of delivery	Free at the point of delivery

*Data for England only available at United Kingdom level i.e. the four countries including England.

#### Availability, provision and access to DSS in the Netherlands, England and Denmark

The primary and secondary documents that we used for the analysis can be found in Additional file 2, and were published between 1999–2012.

**Figure 2:**
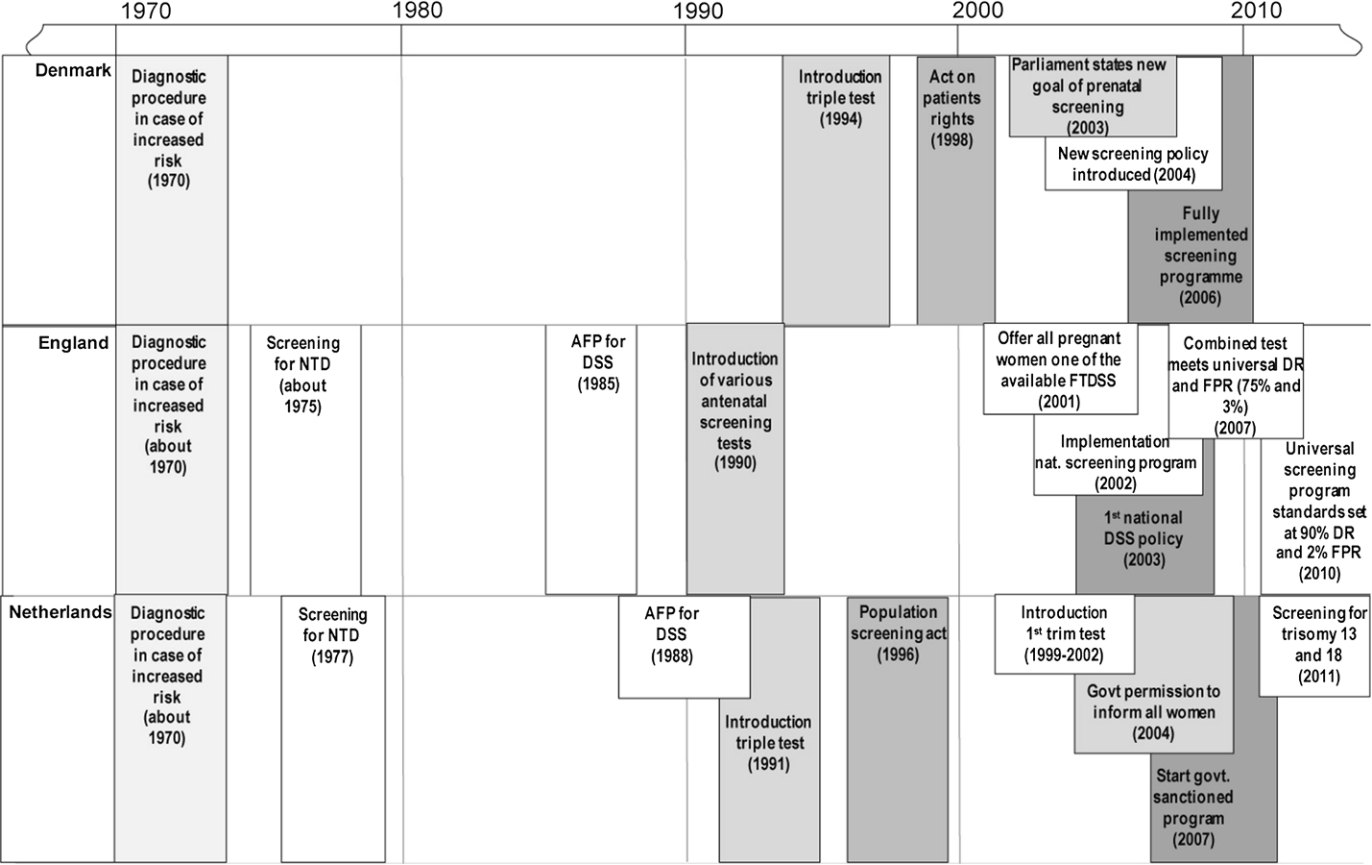
**Timelines of implementation of DSS in Denmark, England and the Netherlands.** *Grey scales indicate identical elements within the national programmes.*

The participating countries all have a current national DSS programme. Figure 2 gives an overview of the timelines by country in terms of DSS provision [34–39, 1]. Introduction of the different programmes and uptake rates have shown a different trend, although uptake increased in all three countries, over time, England and Denmark showed (proportionately) a similar increase (Figure 1) [8, 9, 40–42]. In England, due to a change in policy in 2004 to make DSS available to all pregnant women, all hospitals now have a DSS programme in place. In Denmark the DSS programme was adopted in 2004 and in 2006 all women had access to the programme. In the Netherlands the programme was fully implemented in 2007 [7–9].

In all three countries, it is policy for DSS to be available for all pregnant women, and first-trimester (combined) screening is the standard. An opt-in system is used for participation in DSS [36, 23, 43, 44]. DSS comes within national antenatal healthcare in all the three countries, and while DSS is available from private healthcare companies it is probably used by a small proportion of the pregnant population, although exact figures are unknown.

All three countries provide national information leaflets to pregnant women that show close resemblance on content on terms of informed choice, purpose and performance, knowledge of tested conditions, knowledge of interpretation of (possible) test results, knowledge of further diagnostic options. Information on possibilities if DS is diagnosed was only mentioned in English and Danish leaflets [45–47].

The guidelines for information provision differed between the UK and the Netherlands and Denmark in respect of the ‘right not to know’ [36, 23, 43].

In the Netherlands and Denmark the ‘right not to know’ is explicitly described in legislation regarding prenatal screening. The ‘right not to know’ is founded on an ethical principle which emphasizes patient autonomy and gives patients the right to refuse certain medical information. No such principle is included in screening guidelines or policy in England. Only in the Netherlands women are explicitly asked if they want information on DSS before information was provided [36, 23].

In England and Denmark, DSS is free at the point of delivery for all pregnant women [36, 43]. In the Netherlands DSS is reimbursed only for women 36 years of age and over or those at high prior risk for fetal anomalies; women younger than 36 years of age and with no prior risk are charged a fee of approximately €160,00 when participating in the prenatal screening programme [23, 48].

### Stakeholder interviews: qualitative data

The transcribed interviews were thematically analysed. The identified themes were: value of informed choice, influence on screening choices and the impact of financial considerations [28].

#### The value of informed choice

In all three countries the informed and autonomous choice of women in relation to DSS is enshrined in policy and practice guidelines. During interviews, stakeholders also emphasised these values in the context of screening delivery. For example English stakeholders commented:

*“….there are more people being offered the test, but if the question is, are we encouraging women’s decision one way rather than another, to have a test or not, the answer is definitely not…” (*C2-English, research*).*

*“…we don't want to have an uptake target, because it is about choice....”(*C1- English, policy/ development).

Danish stakeholders agreed with this position:

*“I don’t decide what they want, I just tell them that there is a choice here and then they will actively have to say yes or no*” *(*B2-Danish, professional specialism/research*).*

*“I have spoken to thousands of women……they are very pragmatic and it's also my impression actually that Danish women take this seriously…..even if they have differences in education right now, they seemed to understand the whole aspects of this…” (*B6-Danish, development/research*).*

Dutch stakeholder also recognised the value of informed choice:

*“….being explicitly informed before making a choice is important to consider all the pro’s and con’s before making a choice…..”* (A1-Dutch, policy/development).

*“…women are being counselled in a non-directive way, so they are able to make an individual choice*....”(A4-Dutch, professional specialism).

#### Influences on screening choices

At the same time as agreeing that informed choice was enshrined in policy and delivered in practice, stakeholders acknowledged the influence that others, including healthcare professionals, might have on uptake of DSS

*“…the uptake is very much influenced by what the midwife says to them....and also of what they are hearing from family and friends. Their preconceived ideas and what is being said to them by the midwife is going to be what decides what they do” (*C3-English, professional specialism/ development*).*

*“....the uptake is influenced by the information that is available and how the screening is offered and how it is brought across by the healthcare professional” (*C4-English, development*).*

The countries all followed an ‘opt-in’ system for DSS, however the Danish stakeholders also recognised the social context of antenatal care and the way that the offer was framed influenced the options women appeared to realistically have.

*“…a lot of women make the choice they feel this is reasonable, and they use the fact, that colleagues, their sisters* etcetera *went for screening…I probably think that in practice terms, it is probably an opt-out, whereas it intended to be an opt-in….” (*B6-Danish, development/research*).*

*“….it’s a culture signal…..and you know from your sister when she was pregnant, or your best friend when she was pregnant, how it is a good thing to do….” (*B5-Danish, policy/development).

Danish stakeholders also suggested that a test offered by government could be perceived as a recommendation:

*“…..and if you present an offer.......a woman has to make a decision and it is very hard to say no to something, off course it is like that, so there can be a pressure just by presenting it…” (*B4-Danish, professional specialism/research).

“…[it] *means that some women may think that this is a test recommended by the government and therefore equals ‘this must be good*” (B1-Danish, professional specialism/research).

*“….because the offer is made by the government, there might be people who say I cannot decide, I will go with the flow*....”(B2-Danish, professional specialism/research*).*

In Denmark, the opt in process was seen as important in supporting an active informed choice and helping to protect against just ‘going with the flow’.

*“The woman has to make an active choice of saying I want this screening…..women have to book on the internet or make a phone call…..” (*B1-Danish, professional specialism/research*).*

Some Dutch stakeholders voiced concerns about an over emphasis on informed choice*.*

*“…I sometimes think, we put too much effort on informed choice…..in normal life how many real informed choices do we actually make…..” (*A3-Dutch, professional specialism*).*

In one case this was given as a reason for the relatively low uptake in the Netherlands.

*“….the effort being made on informed choice can be of influence on low uptake rates in the Netherlands….” (*A4-Dutch, professional specialism*).*

In the Netherlands the ‘right not to know’ was explicitly recognised by Dutch stakeholders

“*Before counselling women are asked if they want information on the test” (*A4-Dutch, professional specialism*).*

*“....a woman can indicate that she does not want to know about any prenatal test, then that’s also ok, one shouldn’t overwhelm women if they don’t want that” (*A1-Dutch, policy/development).

On the other hand, it was recognised that enshrining this right in practice may produce a dilemma for the DSS programme and informed choice:

“....*there is a problem attached to it* [the right not to know]....*if you ask somebody if they want to know* [about DSS], *and they don’t, is it clear for the parents what it is about?....do they exactly know about the consequences?” (*A3-Dutch, professional specialism*).*

In Denmark the right not to know also has legal standing but does not appear to be implemented as ‘strongly’ as in the Netherlands. It was recognised again by a Danish stakeholder that the context of an offer affects the way in which this right is perceived.

“....*women have the right not to know, but when you offer the screening you are already sort of pressurizing it*…”(B6-Danish, development/research).

In England this ‘right not to know’ is not stated in legislation, although in practice and in light of the emphasis on informed choice, stakeholders believed that midwives would likely to be sensitive to the wishes of a woman who does not want to discuss DSS any further:

*“So the subject is raised with everybody, if she says, no, I don’t want to hear anything more, then she won’t be told anything more….” (*C2-English, research).

#### Financial considerations and impact on screening choices

Unlike in England and Denmark, in the Netherlands, pregnant women are charged a fee for DSS unless they are older than 36 years. Some Dutch stakeholders believed that this fee has an influence on uptake rates:

*“……For some people the charge of €160,00 could cause distrust. For some people the amount of money has impact on their available budget…..” (*A4-Dutch, professional specialism*).*

*”….in one Dutch region where the test was reimbursed for everybody, the uptake increased by 50%.....but one could question whether these women were able to make an informed choice…” (*A3-Dutch, professional specialism*).*

Others were less confident on the effect of the fee charged:

“.*…money can play a role in decision making, but that is something we don’t know yet” (*A1-Dutch, policy/development*).*

*“....I wonder whether the fee of €160,00 could be a reason to decline the test*.....” (A2-Dutch, development/research)

*“preliminary research showed that costs have not affected the uptake much.....” (*A2-Dutch, development/research*).*

## Discussion

### Summary of results

With this study we considered structural and policy differences between the Netherlands, England and Denmark to explain observed variation in national uptake rates. Except for religion, we found many similarities in demographics. Health and social care policies were also comparable in the three countries. However, the additional cost for DSS over and above standard antenatal care in the Netherlands and an emphasis on the ‘right not to know’ about screening in this country were identified as potential explanations for the ‘low’ uptake rates of DSS in the Netherlands. The social context and positive framing of the offer at the service delivery level may play a role in the relatively high uptake rates in Denmark.

### Population characteristics

Our survey of basic demographics showed, superficially at least, more similarities than differences in population characteristics. Religious affiliation, which has frequently been associated with lower uptake of screening [14, 15] was actually least evident in the Netherlands data. However, whether religious affiliation equates to religious belief is not necessarily the case. For example, Danish citizens are registered as members of the Danish church unless they actively take back their registration. In England, while women may not have a strong religious affiliation they may still, culturally align themselves with the church. This may partly be due to the strong link between the church and the English education system identification, for example. In conclusion, there was no clear explanation that screening uptake rates were closely aligned with population characteristics in the direction that would be predicted by the literature.

### Screening policy and programmes

Our investigation aimed to find some explanations for the wide variation in screening uptake across the three European countries within the way screening programmes were set up and structured. In particular, we wanted to consider the structural factors which may contribute to the Netherlands having a relatively low DSS uptake rate compared to the other nations. We will consider these factors first.

In this study we were able to identify two specific characteristics in the Dutch DSS programme policy which were different to Denmark and England. The first was the ‘strong’ implementation of the right not to know principle, and the second was the charged fee for DSS in women under 36 years old with no history of fetal anomaly.

To understand the context of these two policy points it is important to understand the historical context of DSS in the Netherlands. From 1991 until 2004 an extensive public debate took place between government, professional groups, patient organisations and Health Council. As a result of this, a universal DSS programme promoted by the government was considered to communicate reduced acceptance of disabled individuals. There were fears of ‘genetic cleansing’ via the promotion of abortion to prevent the birth of disabled children. In addition, the direction of the debate was against medicalization of pregnancy and the generation of what was considered unnecessary anxiety in pregnancy [49–53]. With the implementation of the current screening programme, the Dutch government has tried to reach a compromise. By offering DSS to all women equal access was guaranteed, but the restrained policy and the age limit was maintained by the implementation of the ‘right not to know’ and a fee charged [52].

Garcia et al. have stated that the moral significance of prenatal testing is inseparably bound with the social context in which it is practiced [54]. The current Dutch screening programme is an outcome of a large public debate and therefore reflects the social context in which individual decision-making has to take place. Charging a fee for DSS where all other pregnancy related healthcare is covered conveys a message that to have screening is not ‘just’ routine, and thus DSS in the Netherlands has not become normalised in antenatal care as it has, for example, in Denmark [53]. The direct effect of this policy on individual decision making is unclear, but it is part of the context in which Dutch pregnant women make their decisions and could be an explanation of the lower uptake in the Netherlands when compared to countries where universal screening is actively offered to all pregnant women regardless of age, free at the point of delivery.

More difficult to explain from our data is the significant difference in uptake between England and Denmark. One explanation may be the way the offer of DSS is presented in Denmark compared to England. Danish stakeholders acknowledged the role of healthcare professionals and how screening can be perceived as a recommendation from the government, but were also convinced that the ‘opt in’ system prevented patients from making uninformed choices. Evidence for the role of healthcare professionals on screening choices is mixed. Some studies suggest that individual healthcare professionals’ attitudes do not have a significant effect on women’s DSS choices [55, 16]. However, one UK study found that in services where DSS uptake was high, health care professionals as a group held a more positive attitude towards testing than in services where uptake was lower [16]. The authors suggested that *“the observed association between healthcare professionals’ attitudes and uptake rates by hospitals raises the question of whether healthcare professionals’ attitudes might influence systems of care, not just communication with pregnant women”* (p 868)*.* It might be that on the whole, Danish healthcare professionals hold more positive attitudes towards DSS than do their English (or Dutch) counterparts. The Danish stakeholders also recognised the social context of DSS and the way that the offer is framed influences the options women appear to have. In a country where screening uptake has historically been relatively high, women may themselves hold more positive views about DSS and this is likely to have some influence on the choices of other pregnant women in their social milieu [54, 56]. Danish stakeholders comments about Denmark appearing to offer an ‘opt out’ programme, suggest a different frame associated with loss of opportunities (e.g. if everyone else is having it), different from the ‘frame’ of ‘the right not to know’ and costs in the Netherlands. Presenting participation as an opportunity (both opt-in and opt-out) appear to bias decision making and affects uptake. Opportunity frames seem to draw attention on treatment and therefore influence decision making [57]. The Danish perception of an ‘opt out’ programme, could also be a reflection of the context and service delivery. As these are important variables in determining uptake this might play a role in the high uptake rates in Denmark [58]. In England, the emphasize on informed decision making and opt-in, frames the offer in a different way, but the effect of this on uptake rates is less clear. The absence of costs associated with the utilization of testing and emphasize on informed decision making (opportunity framing) might account partly for the English uptake rates [57, 59, 60].

However, these are suppositions and further empirical research is required to address these possible social and cultural differences within national DSS services and the countries within which they are situated.

### Contribution to knowledge and limitations and future research

This paper has made an important preliminary attempt to consider how macro-level demographic, social and healthcare delivery factors may have an impact on national uptake rates for DSS. It has suggested a number of policy level and system characteristics that may go some way to explaining the relatively low uptake rates of DSS in the Netherlands when compared to England and Denmark.

An important limitation of this study was that the primary researchers were Dutch, although they had a good knowledge of English. The use of primary documents often requires knowledge of language of the country. This resulted in potentially less primary data being collected from the Denmark when compared to the other two groups.

## Conclusion

This study aimed to assess the impact of a range of national characteristics in the Netherlands, England and Denmark on DSS uptake rates. Our results indicate that utilization of DSS may be influenced by the way it is being offered at both policy and service delivery level, and that the ‘offer’ is also contextualised within the woman’s social environment. It is proposed that in the Netherlands having to pay for DSS, the public debate that preceded this decision and the emphasis on the right not to know, are in combination likely to explain the relatively low uptake of DSS. In contrast, a social context where screening is viewed as routine and the offer framed possibly, if not strongly, as a recommendation may play a role in the relatively high uptake of DSS in Denmark. Future research can build on this preliminary work to assess empirically the effect of different aspects of national policies and healthcare culture on individual decision making. Such work is likely to require quantitative, qualitative, and observational approaches.

## Electronic supplementary material

Additional file 1: **Socio-demographic population profiles, cultural factors, organisation of healthcare and social system.** (DOCX 14 KB)

Additional file 2: **Primary and Secondary sources macro overview of the delivery of national screening programmes.** (DOCX 22 KB)
