# Supplementary material for: Explaining variation in Down’s syndrome screening uptake: comparing the Netherlands with England and Denmark using documentary analysis and expert stakeholder interviews
Source: BMC Health Serv Res. 2014 Sep 25;14:437. doi: 10.1186/1472-6963-14-437 (PMC4263059; doi:10.1186/1472-6963-14-437)
Supplement: Supplementary file 1 — Additional file 1: Socio-demographic population profiles, cultural factors, organisation of healthcare and social system. (DOCX 14 KB) [file 12913_2014_3522_MOESM1_ESM.docx]

**Additional file 1**

**Socio-demographic population profiles, cultural factors, organisation of healthcare and social system**

(1) European Statistical System (2012): Crude birth rate per 1000 inhabitants. <http://epp.eurostat.ec.europa.eu/tgm/table.do?tab=table&init=1&plugin=0&language=en&pcode=tps00112>.

(2) CBS Statline(2010): Birth; key figures. http://statline.cbs.nl/StatWeb/publication/?DM=SLEN&PA=37422ENG&D1=41&D2=0,10,20,30,40,50,(l-3)-l&LA=EN&VW=T.

(3) Statbank Denmark(2010): Average age of women given birth for the first time. <http://www.statbank.dk/FOLK1>.

(4) Office for national statistics(2010): Live births in England and Wales by characteristics of mother 2010. <http://www.ons.gov.uk/ons/rel/vsob1/characteristics-of-Mother-1--england-and-wales/2010/stb-live-births-in-england-and-wales-by-characteristics-of-mother-2010.html>.

(5) CBS Statline (2009): Participation in social activities. <http://statline.cbs.nl/StatWeb/publication/?DM=SLEN&PA=60027eng&D1=0,6,10,59-70,91-96&D2=0-2&D3=8-13&LA=EN&HDR=G2&STB=T,G1&VW=T>.

(6) Statbank Denmark(2009): Population and membership of national church. <http://www.statbank.dk/BEV21>.

(7) Dobbs J, Green H, Zealey L(2006): Focus on religion and ethnicity.;National Statistics.

(8) European Commission: Migrants in Europe A statistical portrait of the first and second generation. Luxembourg: Publications Office of the European Union. 2011.

(9) European Commission: Key data on Education in Europe 2009. 2009, EACEA P9 Eurydice.

(10) Eurostat(2010): Gross domestic product per capita in purchasing power standards (PPS). <http://epp.eurostat.ec.europa.eu/tgm/table.do?tab=table&init=1&plugin=1&language=en&pcode=tec00114>.

(11) van der Zee J, Kroneman MW: Bismarck or Beveridge: a beauty contest between dinosaurs. BMC Health Serv Res 2007, 26;7:94.

(12) Ministry of Health, Welfare and Sport: Equal Treatment of Disabled and Chronically Ill People Act. 2003. Dutch translation: Wet gelijke behandeling op grond van handicap of chronische ziekte (WGBH/CZ)

(13) Danish Parliament: *B 43, Folketingsbeslutning om ligestilling og ligebehandling af handicappede med andre borgere.* Parliamentary resolution on equalisation of opportunities and equal treatment of persons with disabilities. 1993.

(14) Parliament of the United Kingdom: Equality Act 2010. 2010.

(15) Ministry of Health, Welfare and Sport: Law on general insurance against special medical expenses. Dutch translation: Algemene Wet Bijzondere Ziektekosten (AWBZ). 1967.

(16) Ministry of Social Affairs and Employment: Regeling tegemoetkoming ouders van thuiswonende gehandicapte kinderen (TOG) 1999.

(17) The Ministry of Social Affairs and Integration: Social policy in Denmark . 2012.

(18) Ministry of Health, Welfare and Sport: Social Support Act. Dutch translation: Wet maatschappelijke ondersteuning. 2006.

(19) Boyd PA, Devigan C, Khoshnood B, Loane M, Garne E, Dolk H, and the EOROCAT working group: Survey of prenatal screening policies in Europe for structural malformations and chromosome anomalies, and their impact on detection and termination rates for neural tube defects and Down's syndrome. BJOG 2008, 115(6):689-696.

(20) Lovitidende for Kongeriget. Law No. 350 of 13 June 1973 on the interruption of pregnancy. 1973.

(21) Ministry of Health, Welfare and Sport: Law on the interruption of pregnancy. Dutch translation: Wet afbreking zwangerschap. 1981.

(22) Garne E, Khoshnood B, Loane M, Boyd P, Dolk H, EUROCAT Working Group: Termination of pregnancy for fetal anomaly after 23 weeks of gestation: a European register-based study. BJOG 2010 May;117(6):660-666.
