# Supplementary material for: Explaining variation in Down’s syndrome screening uptake: comparing the Netherlands with England and Denmark using documentary analysis and expert stakeholder interviews
Source: BMC Health Serv Res. 2014 Sep 25;14:437. doi: 10.1186/1472-6963-14-437 (PMC4263059; doi:10.1186/1472-6963-14-437)
Supplement: Supplementary file 2 — Additional file 2: Primary and Secondary sources macro overview of the delivery of national screening programmes. (DOCX 22 KB) [file 12913_2014_3522_MOESM2_ESM.docx]

**Additional file 2**

**Primary and Secondary sources macro overview of the delivery of national screening programmes**

**The Netherlands**

Gezondheidsraad: Commissie WBO. Wet bevolkingsonderzoek: prenatale screening en risicoperceptie. Den Haag: Gezondheidsraad, 1999; publicatie nr 1999/04WBO.

Gezondheidsraad: Prenatale screening: Downsyndroom, neuralebuisdefecten, routineechoscopie. Den Haag: Gezondheidsraad, 2001; publicatie nr 2001/11.

Gezondheidsraad: Wet bevolkingsonderzoek: screening op downsyndroom vroeg in de zwangerschap. Den Haag: Gezondheidsraad, 2003; publicatie nr 2003/01WBO.

Gezondheidsraad: Prenatale screening (2); Downsyndroom, neurale buis defecten. Den Haag: Gezondheidsraad, 2004; publicatie nr 2004/06.

Gezondheidsraad: Commissie WBO. Wet bevolkingsonderzoek: aanzet tot een landelijk programma voor prenatale screening; downsyndroom en neurale buis defecten. Den Haag: Gezondheidsraad, 2006; publicatie nr 2006/03WBO.

Gezondheidsraad: Wet bevolkingsonderzoek: prenatale screening op downsyndroom en neurale buis defecten. Den Haag: Gezondheidsraad, 2007; publicatienr. 2007/05WBO.

Gezondheidsraad: Wet bevolkingsonderzoek: prenatale screening op trisomie 13 en 18. Den Haag: Gezondheidsraad, 2010; publicatienr. 2010/01WBO.

Wet op de beroepen in de individuele gezondheidszorg (Wet BIG).

Wet op de geneeskundige behandelingsovereenkomst (WGBO).

Wet bevolkingsonderzoek (WBO).

RIVM -Centraal Orgaan. Draaiboek prenatale screening Downsyndroom en Structureel Echoscopisch Onderzoek versie 2.0. 2011.

RIVM -Centraal Orgaan. Kwaliteitsborging prenatale screening door regionale centra-voortgangsrapportage plan van aanpak. 2012 .

RIVM -Centraal Orgaan. Posttest counseling bij een verhoogde kans uitslag bij de combinatietest. 2012.

RIVM -Centraal Orgaan. Algemene kwaliteitseisen counselors. versie 4.2011.

RIVM -Centraal Orgaan. Kwaliteitseisen voor het geven van de uitslag. versie 2. 2011.

RIVM -Centraal Orgaan. Landelijke kwaliteitseisen informed consent en privacy binnen de prenatale screening op Downsyndroom en het SEO. versie 2. 2011.

RIVM -Centraal Orgaan. Landelijke opleidingseisen aan de counseling. 2011.

RIVM -Centraal Orgaan. Overeenkomst Regionaal Centrum Prenatale Screening en praktijk voor counseling . 2011.

RIVM -Centraal Orgaan. Kwaliteitsovereenkomst tussen het regionaal centrum en counselors die in het kader van de prenatale screening de counselinggesprekken uitvoeren. 2011.

Schielen PCJI, Elvers LH, Loeber JG: Risk estimation for Down's syndrome and neural tube defects by analysis of triple test parameters in maternal serum 1999-2002 .2004. RIVM rapport 230041002.

Schielen PCJI, Elvers LH, Loeber JG: Down syndroom risicoschatting in het 1e trimester door bepaling van PAPP-A en vrij beta-hCG in serum. 2003. RIVM rapport 199101008.

RIVM -Centraal Orgaan: Tarieven en declaraties voor de screening op Downsyndroom en het SEO. 2011.

Wortelboer EJ, Koster MP, Stoutenbeek P, Loeber JG, Visser GH, Schielen PC: Fifteen years of triple tests in The Netherlands; the life cycle of a screening test. Prenat Diagn 2008, 28(10):950-955.

RIVM -Centraal Orgaan: Patient leaflet: Informatie over de screening op Downsyndroom 2011. www.rivm.nl/zwangerschapsscreening.

**England**

Clinical Genetics Society Prenatal Screening Group: Clinical Genetics and Antenatal/ Fetal Medicine: Liaison and Training (Report) 2008 (http://www.clingensoc.org).

NHS Antenatal and Newborn Screening Programmes: National Down’s Syndrome Screening Programme for England. Antenatal Screening -Working Standards for Down’s Syndrome Screening 2007. 2007.

NHS UK National Screening Committee Programmes Directorate: National Down’s Syndrome screening programme for England-A Handbook for Staff 2004.

National Collaborating Centre for Women’s and Children’s Health Commissioned by the National Institute for Health and Clinical Excellence: Antenatal care Routine care for the healthy pregnant woman 2010.

NHS Fetal Anomaly Screening Programme: Remits, Aims and Objectives. 2009.

NHS Fetal Anomaly Screening Programme: Testing for Down’s syndrome in pregnancy Patient leaflet. 2010.

NHS UK National screening Committee: Screening tests for you and your baby. 2012.

Abortion Act 1967 CH. 87.

Skirton H, Barr O: Antenatal screening and informed choice: across-sectional survey of parents and professionals. Midwifery 2010, 26(6):596-602.

The Down’s Syndrome Association: What can you claim? A basic guide to benefits which people with learning disabilities may be able to claim. 2010.

The Down’s Syndrome Association: Working Tax Credit and Child Tax Credit. 2010.

De Souza E, Alberman E, Morris JK: Down’s syndrome: screening and antenatal diagnosis regionally in England and Wales 1989–2008. J Med Screen 2010, 17:170-175.

NHS Fetal Anomaly Screening Programme: DQASS Down’s Syndrome Screening Quality Assurance Support Service - Working structure. 2012.

NHS Fetal Anomaly Screening Programme: Screening for Down’s syndrome: UK NSC Policy recommendations 2007-2010: Model of Best Practice. 2008.

NHS Fetal Anomaly Screening Programme: Screening for Down’s syndrome: UK NSC Policy recommendations 2011–2014 Model of Best Practice 2011.

Department of Health: The Handbook to the NHS Constitution – for England. 2012.

Rowe R, Puddicombe D, Hockley C, Redshaw M: Offer and uptake of prenatal screening for Down syndrome in women from different social and ethnic backgrounds. Prenat Diagn 2008, 28: 1245-1250.

Department of Health: Our Inheritance, our future: Realising the potential of genetics in the NHS. 2003.

NHS Fetal Anomaly Screening Programme: Remits, Aims and Objectives 2009.

Commission for Healthcare Audit and Inspection: Towards better births: A review of maternity services in England. 2008.

**Denmark**

Ekelund CK, Petersen OB, Skibsted L, Kjaergaard S, Vogel I, Tabor A, Danish Fetal Medicine Research Group: First-trimester screening for trisomy 21 in Denmark: implications for detection and birth rates of trisomy 18 and trisomy 13. Ultrasound Obstet Gynecol 2011, 38(2):140-144.

Gross ML. Abortion and Neonatocide: Ethics, Practice and Policy in four nation. Bioethics 2002, 16(3): 202-230.

Schwennesen N, Nordahl Svendsen M, Koch L: Beyond informed choice: prenatal risk assessment, decision-making and trust. Etikk i praksis Nordic Journal of Applied ethics 2008, 1:11-31.

Garne E, Berghold A, Johnson Z, Stoll C: Different Policies on Prenatal Ultrasound Screening Programmes and Induced Abortions Explain Regional Variations in Infant Mortality with Congenital Malformations. Fetal Diagn Ther 2001, 16(3):153-157.

Tabor A, Vestergaard CHF, Lidegaard Ø: Fetal loss rate after chorionic villus sampling and amniocentesis: an 11-year national registry study. Ultrasound Obstet Gynecol 2009, 34: 19-24.

Sundhedsstyrelsen og komiteen for Sundhedsoplysning: Risikovurdering og fosterdiagnostik : information til gravid 2004.

Ministry of Health and prevention: Health Care in Denmark.

Ekelund CK, Jorgensen FS, Petersen OB, Sundberg K, Tabor A: Danish Fetal Medicine Research Group. Impact of a new national screening policy for Down's syndrome in Denmark: population based cohort study. BMJ 2008: 27;337:a2547.

Wøjdemann KR, Shalmi AC, Christiansen M, Larsen SO, Sundberg K, Brocks V, Bang J, Nørgaard-Pedersen, Tabor A: Improved first-trimester Down syndrome screening performance by lowering the false-positive rate: a prospective study of 9941 low-risk women. Ultrasound Obstet Gynecol 2005, 25: 227-233.

Kirkegaard I, Petersen OB, Uldbjerg N, Tørring N: Improved performance of first-trimester combined screening for trisomy 21 with the double test taken before a gestational age of 10 weeks. Prenat diagn 2008, 28(9):839-844.

Dahl K, Kesmodel U, Hvidman L, Olesen F: Informed consent: attitudes, knowledge and information concerning prenatal examinations. Acta Obstet Gynecol Scand 2006, 85(12):1420-5.

Vestergaard CHF, Lidegaard Ø, Tabor A: Invasive prenatal diagnostic practice in Denmark 1996 to 2006.Acta Obstet Gynecol Scand 2009, 88(3): 362-365.

Christiansen T: Organization and financing of the Danish health care system. Health Policy 2002, 59(2):107-18.

Prenatal examinations: UndersØgelser af det ufØdte barn-engelsk Komiteen for Sundhedsoplysning 2005.

Sundhedsstyrelsen: Fosterdiagnostik og risikovurdering. Rapport 2003.

Sundhedsstyrelsen: Retningslinjer for fosterdiagnostik- prænatal information, risikovurdering, rådgivning og diagnostik 2004.

Schiøtt KM, Christiansen M, Petersen OB, Lind Sørensen T, Uldbjerg N: The “Consecutive Combined Test”—using Double Test from week 8 + 0 and Nuchal Translucency Scan, for first trimester screening for Down Syndrome. Prenat Diagnosis 2006, 26(12):1105-1109.

Vrangbaek K. The Danish Health Care System. In: Thomson, Sarah et al. (eds.): International Profiles of Health Care Systems, 2012: Australia, Canada, Denmark, England, France, Germany, Iceland, Italy, Japan, the Netherlands, New Zealand, Norway, Sweden, Switzerland, and the United States. New York and Washington, The Commonwealth Fund,2012; 26-31.
